# Editorial: Intensive care unit acquired weakness: potential role of medical nutrition treatment quantity, timing, and composition

**DOI:** 10.3389/fnut.2023.1295911

**Published:** 2023-11-24

**Authors:** Natalija Vukovic, Rémy Meier, Agnieszka Guligowska, Polina Zalizko

**Affiliations:** ^1^University Clinical Center Nis, Clinic for Anesthesiology, Reanimation and Intensive Care, Nis, Serbia; ^2^AMB - Medical Practice MagenDarm, Basel, Switzerland; ^3^Department of Geriatrics, Medical University of Lodz, Łódz, Poland; ^4^Department of Internal Medicine, Pauls Stradins Clinical University Hospital, University of Latvia, Riga, Latvia

**Keywords:** muscle weakness, nutrition therapy, critical illness, critical illness polyneuropathy, myopathy, muscular atrophy, ventilator weaning

## Introduction

Profound muscle weakness during and after critical illness for which there is no other clinical explanation is termed intensive care unit acquired weakness (ICUAW) (1). Some of the terms used for the ICUAW syndrome are intensive care unit acquired paresis, syndrome of acquired weakness (2), critical illness myopathy (CIM), critical illness polyneuropathy (CIP), critical illness polineuromyopathy (3), or critical illness neuromyopathy (CINM) (4). According to Krammer (4), besides CIM, CIP, and CINM, the term ICUAW includes muscle atrophy too.

## Epidemiology

As stated by De Jonghe et al. (5), the clinical incidence of ICUAW is 25.3%. Nonetheless, different data can be found in the literature and they mainly differ according to the type of acute illness and diagnostic criteria used (Czapla et al.).

## Pathology

The primary pathophysiological finding in peripheral nerves of ICUAW patients is axonal degeneration without demyelination (6). In the muscle, there is prominent myonecrosis with the loss of myosin filaments, and a change in actin: myosin ratio (7), disruption of muscle organization (8), vacuolization, and phagocytosis of myofilaments.

## Clinical presentation

Clinical presentations of ICUAW differ in the degree of reduction of motor and sensory action potentials. In some patients, loss of deep tendon reflexes, as well as, loss of sensitivity to pain, temperature, and vibration can be present.

The most frequently affected muscles are limb muscle groups and respiratory muscles, thus flaccid quadriparesis and neuromuscular respiratory failure can be seen. Prolonged mechanical ventilation and difficult weaning are present (9) and there is susceptibility to recurrent respiratory infection and nosocomial pneumonia. Ophthalmoplegia and facial weakness may rarely develop (10).

## Diagnostics

There are various diagnostic tests that explore different pathophysiological and clinical aspects of ICUAW syndrome.

The Medical Research Council (MRC) sum score is used as a screening tool for ICUAW. This score evaluates muscle strength in three upper and lower limb muscle groups and assesses it from 0 to 5. The maximal score is 60 and if it is <48 there is diagnostically important muscle weakness (11). Its major limitations are that the patient has to be awake and cooperative and there is a substantial interobserver measure difference.

Several ICUAW diagnostic tests have a peripheral neuron-muscle connection as the examination goal. In this way, hand grip dynamometry and direct muscle stimulation (peroneal nerve test) can diagnose CIP (11). The former needs special expertise which would present its major limitation.

Electromyography and nerve conduction studies can be used in uncooperative patients (12). These tests can determine ICUAW early during critical illness (10, 11) and more importantly, distinguish this syndrome from other diagnoses.

Two diagnostic tests explore muscles on different levels of comprehension and assumption. Muscle ultrasound is a method with good interobserver reliability and accentuated analysis of muscle architecture. According to Hadda et al. (13), patients with ICUAW have a significantly higher decline in muscle thickness.

Muscle biopsy is the gold standard for diagnosing muscle involvement and differential diagnosis between ICUAW and other diagnoses. Percutaneous muscle biopsy is an easy-to-perform bedside technique (10).

Biochemical markers such as urine nitrogen, blood urea to creatinine, and blood urea to albumin level are markers of nutritional status, dehydration, and liver and renal function and they can help in the assessment of ICUAW clinical presentation (Cai et al.).

## Risk factors

The main risk factors of ICUAW are severe sepsis, the duration of mechanical ventilation, and multiorgan failure. Identification of risk factors can help in defining prediction models for ICUAW (Yang et al.).

Female and older critically ill patients are more prone to develop ICUAW (5).

One of the most important correctible risk factors is hyperglycemia. Strict control of hyperglycemia without hypoglycemia reduces ICUAW incidence (4).

Drugs researched within the development of ICUAW syndrome are corticosteroids, muscle relaxants, sedatives, and some antimicrobial agents (12).

Immobilization is a risk factor too, since, as soon as 4 h after initiating it, muscle degeneration can be observed (3).

## Prevention and treatment

The main intervention in ICUAW treatment nowadays is prevention.

Hyperglycemia correction presents one of the proven preventive strategies (14).

The data are not so clear for the medical nutrition therapy and its relation to autophagy. Namely, tolerating substantial macronutrient deficit early in the critical illness, according to the study of Hermans et al. (15) allowed more efficient activation of autophagy. In this way, late PN, after 7 days in the ICU, initiates faster recovery in patients and reduces weakness. Aminoacid administration suppresses autophagy in muscle which has been shown to be the contributing mechanism in the ICUAW development (15). All of these results differ from the data on the importance of reaching caloric (Lv et al.) and protein goals (Zhu et al.) between the third and the seventh day of critical illness. Therefore, further scientific approval is needed. These studies also have to determine the importance of initiating medical nutrition therapy in the course of critical illness, both enteral (Wang et al.) and parenteral (Li et al.).

Stimulated ketogenesis and supplementation of 3-hydroxybutyrate showed encouraging results in muscle protection from muscle weakness in mice models (16). This might be a novel metabolic strategy.

Reducing the use of sedative drugs and neuromuscular relaxants is a recommended approach for preventing ICUAW (17). The implementation of “sedation holidays” and daily pauses in sedative infusions has been linked to shorter mechanical ventilation and reduced ICUAW development.

Early progressive gradual mobilization is an effective ICUAW treatment strategy. It is associated with reduced mechanical ventilation time, lower incidence of ICUAW, and improved functional outcomes. Together with previously mentioned preventive strategies it might present an aspect of a multifactorial interventional set for improving outcomes for ICUAW patients.

## Outcomes

Intensive care unit-acquired weakness increases acute morbidity, health care costs, and 1-year mortality (18). Prolonged mechanical ventilation and long-term disability are determined characteristics of the outcome of patients with ICUAW (11, 13). The recovery period after ICUAW may last long after the ICU discharge (19). Future studies in this area will change the current perspective regarding the time of initiation and the time of reaching the goals of clinical nutrition (Lopez-Delgado et al.) and possible special nutrients.

## Conclusion

To decrease the occurrence of ICUAW, it is important to follow several prevention strategies. Those are avoiding high blood sugar levels and delaying parenteral nutrition therapy until after 7 days in the ICU. Probably, another promising approach is to minimize sedation and encourage early progressive mobilization. Studies about the activation of ketogenesis and about the use of ketones as special nutrients will add new insight to this scientific area.

## Author contributions

NV: Writing—review & editing, Conceptualization. RM: Supervision, Writing—review & editing. AG: Writing—review & editing. PZ: Investigation, Resources, Writing—original draft.
